# A split ubiquitin system to reveal topology and released peptides of membrane proteins

**DOI:** 10.1186/s12896-017-0391-0

**Published:** 2017-09-02

**Authors:** Qiu-Ping Li, Shuai Wang, Jin-Ying Gou

**Affiliations:** Institute of Plant Biology, School of Life Sciences, Fudan University, Shanghai, 200438 China

**Keywords:** Split ubiquitin, Membrane protein, Extracellular terminus, Cytoplasmic terminus, Released peptide

## Abstract

**Background:**

Membrane proteins define biological functions of membranes in cells. Extracellular peptides of transmembrane proteins receive signals from pathogens or environments, and are the major targets of drug developments. Despite of their essential roles, membrane proteins remain elusive in topological studies due to technique difficulties in their expressions and purifications.

**Methods:**

First, the target gene is cloned into a destination vector to fuse with C terminal ubiquitin at the N or C terminus. Then, Cub vector with target gene and Nub^WT^ or Nub^G^ vectors are transformed into AP4 or AP5 yeast cells, respectively. After mating, the diploid cells are dipped onto selection medium to check the growth. Topology of the target protein is determined according to Table 1.

**Results:**

We present a split ubiquitin topology (SUT) analysis system to study the topology and truncation peptide of membrane proteins in a simple yeast experiment. In the SUT system, transcription activator (TA) fused with a nucleo-cytoplasmic protein shows strong auto-activation with both positive and negative control vectors. TA fused with the cytoplasmic end of membrane proteins activates reporter genes only with positive control vector with a wild type N terminal ubiquitin (Nub^WT^). However, TA fused with the extracellular termini of membrane proteins can’t activate reporter genes even with Nub^WT^. Interestingly,TA fused with the released peptide of a membrane protein shows autoactivation in the SUT system.

**Conclusion:**

The SUT system is a simple and fast experimental procedure complementary to computational predictions and large scale proteomic techniques. The preliminary data from SUT are valuable for pathogen recognitions and new drug developments.

**Electronic supplementary material:**

The online version of this article (10.1186/s12896-017-0391-0) contains supplementary material, which is available to authorized users.

## Background

The functions of biological membranes are mostly determined by membrane proteins, which give each type of membrane its characteristic functional properties. 20% to 30% of the whole human genome proteins are predicted as membrane proteins and 6, 718 are confirmed in a proteomic research [1]. Overall, membrane proteins play critical roles to catch energy, deliver nutrients, detecting and conveying environmental signals into cells. Membrane proteins typically account for 50% of the mass in the plasma membrane and are associated with the lipid bilayers in various ways e.g. transmembrane (TM), hydrophobic binding, lipid anchored proteins and protein-protein interaction proteins [2].

Some TM proteins are cleaved to release small peptide(s) during maturation, translocation or signal transduction. Transit signal (TS) peptides, one kind of typical released peptide with 5 ~ 30 amino acids in length, are found in most type I membrane proteins, recognized by signal-recognition particles and cleaved form mature proteins before they carry out biological functions in correct organelles [3]. A broad spectrum strip rust resistance protein, *Wheat Kinase START*1 (*WKS*1, or *Yr*36), undergoes N terminal truncation and then transportation into the chloroplast to phosphorylate targets which accelerates cell death to confer disease resistance [4, 5]. Chloroplast transit peptides (cTP, ~2kd in length) are reported in 86% of chloroplast proteins, and play crucial roles for the accumulation of proteins in chloroplast to carry out a wide range of metabolic functions [6]. Some released peptides are also involved in cell signaling. 5 ~ 10% GPCRs contain signal peptides in N termini, which regulate receptor densities in the PM and their dimerization in the ER upon truncation [7]. Hence, studies of released peptides are conductive to acquire novel knowledge for the biological functions of cleavable signal peptide and develop new drugs.

A split ubiquitin yeast two hybrid system (MbYTH) is a sensitive and user-friendly system widely used to study membrane protein-protein interactions in yeast [8, 9]. In MbYTH, the ubiquitin is spited into N (Nub) and C terminal (Cub) halves to fuse with bait and prey, respectively [9]. A transcription activator (TA hereafter, LexA-VP) is fused with the target protein by a Cub to form target-Cub-TA fusion protein and could be released by Ubiquitin specific protease (USP) to activate reporter genes if there is an interaction between the bait and prey proteins [8]. The positive control, Nub^WT^, has high binding affinity with Cub and will activate reporter genes independent of protein binding on targets. The negative control, Nub^G^, has an Ile13Gly mutation and low affinity with Cub to avoid spontaneous reassembling (auto activation) [8]. In an interaction landscape of membrane-protein co-purifying complexes in yeast, the protein-protein interactions (PPI) are successfully confirmed by MbYTH [10]. To study the membrane PPIs in the model plant Arabidopsis, 3286 distinct proteins are screened with MbYTH and 12,102 membrane/signaling protein interactions are identified [11]. Therefore, MbYTH is a powerful tool to discover novel interacting membrane protein complexes both in yeast, plants and animals.

Understanding of the topology of membrane proteins is the first step towards their structural elucidations and functional investigations, as well as developments of potential new drugs, as more than half of known drugs target membrane proteins [12]. Cell surface protein could be labeled by sulfo-NH-SS-Biotin and analyzed based on mass data to detect domains outside of the plasma membrane [13, 14]. A cysteine-reactive molecule, maleimide polyethylene glycol (mPEG), is applied to determine the topology of a transmembrane protein by labeling the introduced cysteine in the cytosol [15]. Although these above methods are very powerful for large scale analysis, the detection of a particular protein may be restricted by the efficiency of labeling and enrichment. What is more, after gathering the whole proteome information, most labs will select several promising targets and conduct in depth researches.

There are a variety of programs to predict transmembrane protein topologies according to the physicochemical properties of their amino acids and the “positive inside” rule [16]. These prediction methods use either single sequence information or multiple sequence alignments information, however, their ratios of correctly predicted transmembrane helices are usually under 90% [17]. We also tested several known transmembrane proteins, e.g. VKOR, PIP1;2, GPR3 and HPN, with nine widely used programs, but all of them gave one or more inaccurate results (Table 1) [18–21]. Thus, to study the topological study of a particular membrane protein, a simple but accurate experimental procedure remains essential to develop.

**Table 1:** Summary of computational annotations and reported topologies of known membrane and soluble proteins

	WKS1		VKOR		PIP1;2		HPN		GPR3		GUS	
Gene ID	KT834954.1		NM_119742.4		NM_130159		X07732.1		NM_005281		AJ298139.1	
	N	C	N	C	N	C	N	C	N	C	N	C
TMHMM	no TMHs		*in*	*in*	in	in	in	out	out	in	no TMHs	
SOSUI	no TMHs		No results		No results		in	out	No results		no TMHs	
TOPCONS	no TMHs		*in*	out	in	in	*no TMHs*		out	in	no TMHs	
PHILIUS	no TMHs		*in*	out	in	in	*no TMHs*		*in*	in	no TMHs	
HMMTOP	no TMHs		*in*	out	in	in	in	out	out	in	no TMHs	
TMPRED	no TMHs		out	*in*	in	in	*out*	out	*in*	*out*	*out*	*in*
POLYPHOBIUS	no TMHs		*in*	out	*out*	in	in	out	out	in	no TMHs	
HMM–TM	in	*out*	*in*	out	*out*	in	in	out	out	*out*	no TMHs	
OCTOPUS	no TMHs		*in*	out	in	in	*no TMHs*		out	*out*	no TMHs	
SUT	in	in	out	out	in	in	in	out	out	in	Soluble	
Reference			out	out	in	in	in	out	out	in	Soluble	

N, N terminus. C, C terminus. *Italic*, different from experimental results. Computational predictions were done in versions of December 2016

In the present work, we present a split ubiquitin membrane topology study (SUT) system to collect experimental topology data of membrane proteins in yeast with a new N terminal fusion vector constructed here and also some vectors from the split ubiquitin yeast two hybrid system [22]. In yeast, TA fused with the cytoplasmic termini of TM proteins activates reporter genes with the high affinity Nub^WT^. However, TA fused with the extracellular termini failed to activate reporter genes even with Nub^WT^. TA fused with a released peptide of membrane protein shows auto activation in SUT (Table 2). This approach has the advantage of fast and inexpensive to provide preliminary topology information of a membrane protein.

**Table 2:** Prediction of yeast growth on SD/−L-W/−H/−A medium in SUT system with different types of proteins

Type	Terminus	Nub^G^	Nub^WT^
Nucleo-cytoplasmic protein	TA-Target	Yes^a^	Yes
	Target-TA	Yes^a^	Yes
N terminal truncated membrane protein	TA-Target	Yes^a^	Yes
	Target-TA	No	Yes^c^/No^e^
Membrane protein without truncation	TA-Target	No	Yes^c^/No^e^
	Target-TA	No	Yes^c^/No^e^

Note. ^a^ autoactivation, ^c^ cytoplasmic termini, ^e^ extracellular termini

## Results

### A nucleo-cytoplasmic protein shows autoactivation in the SUT system

**Fig. 1:**
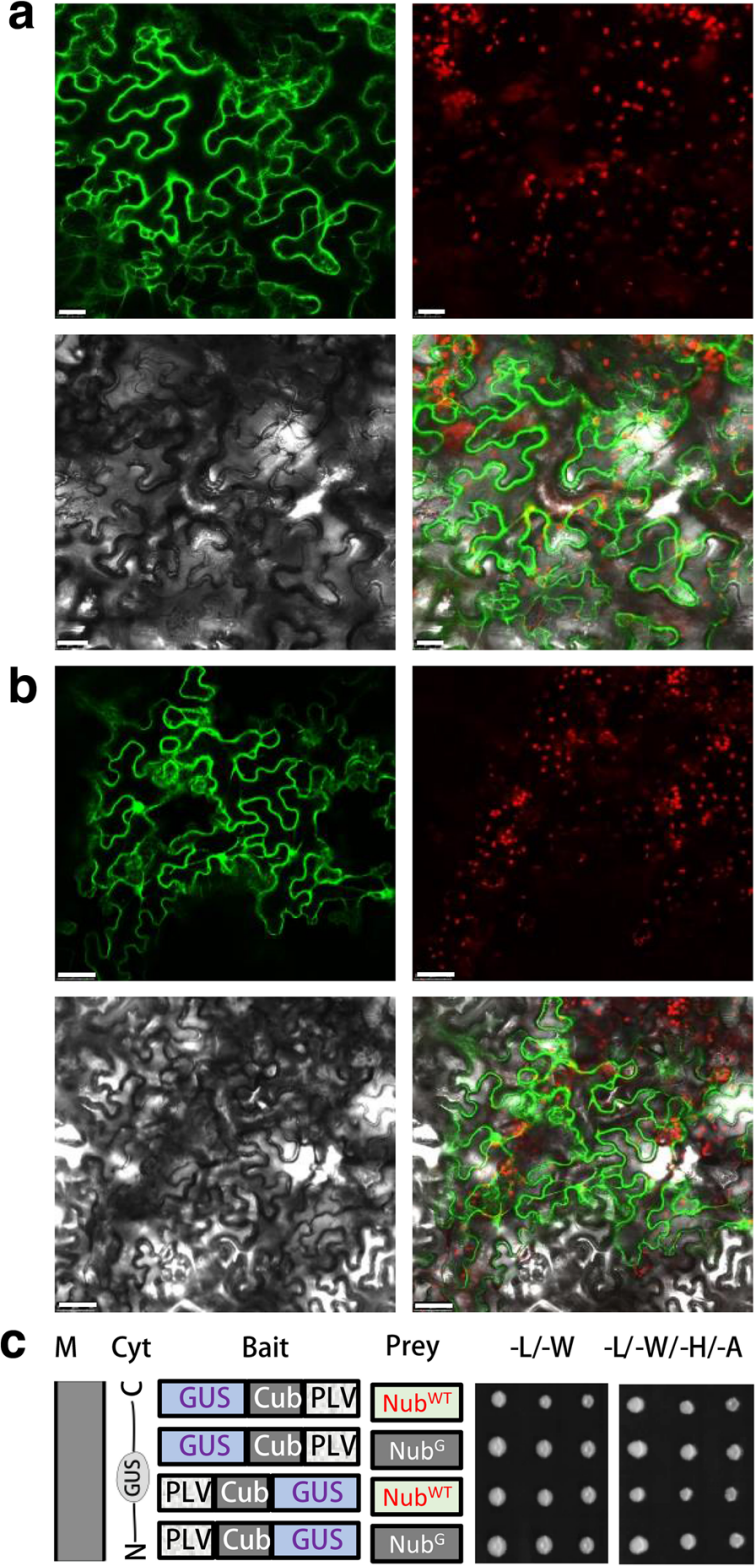
Subcellular localization and yeast growth of a typical nucleocytoplasmic protein. **a**-**b** Confocal micrographs of GUS-GFP (**a**) or GFP-GUS (**b**) fusion proteins in tabaco epidermal cells. The panels from left to right stand for: GFP (green), chloroplast auto-fluorescence (red), cell shape under bright light and overlay of the three panels. Bar stands for 25 μm. GFP, green; chloroplast autofluorescence, red (Chl); cell shape, bright light; and overlay of the three panels. **c** Yeast growth of GUS in SUT system. M, plasma membrane; Cyt, cytoplasm; Cub, C terminal ubiquitin; Nub^WT^, wild type N terminal ubiquitin; Nub^G^, low affinity N terminal ubiquitin with Ile13Gly mutation

To validate the accuracy of the SUT system, a small soluble protein (GUS) is checked for auto-activation. In tobacco leaf epidermal cells, the fluorescence of GUS-GFP and GFP-GUS fusion proteins are detected both in cytoplasm and in the nucleus, a typical phenomenon for nucleo-cytoplasmic localization proteins (Fig. 1a, b). In yeast, TA fused to both N or C terminus of GUS activates reporter genes in combination with both Nub^G^ and Nub^WT^, demonstrating that a nucleo-cytoplasmic localization protein has strong auto-activation in SUT system (Fig. 1c).

### Performance of the SUT system for membrane proteins

**Fig. 2:**
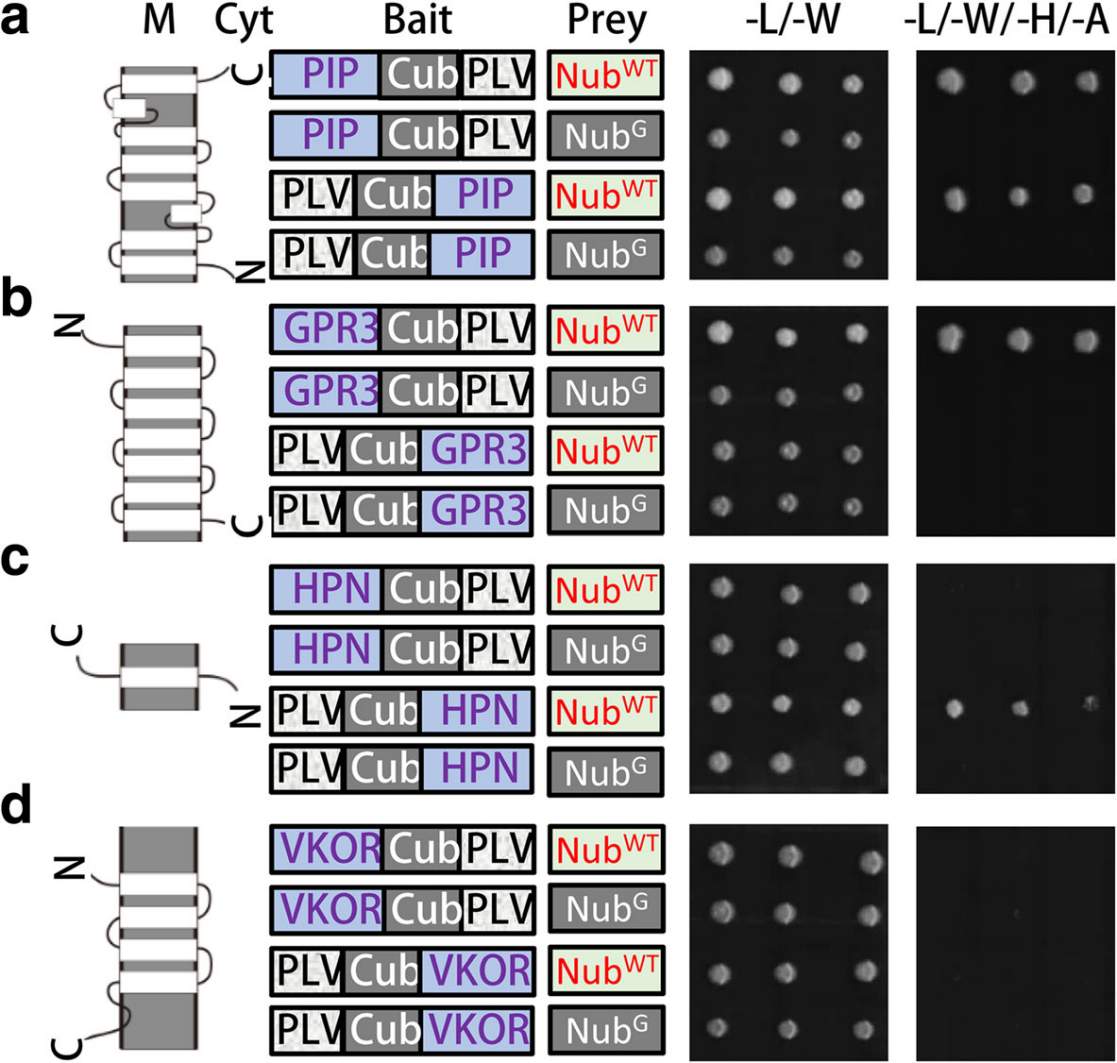
Performance of SUT on four typical membrane proteins with different topologies. **a**-**d** Yeast growth of PIP (**a**), GPR3 (**b**), HPN (**c**), and VKOR (**d**) in the SUT system

In order to further clarify that the SUT system could distinguish membrane proteins, we applied this system to study four known transmembrane proteins, e.g. HPN (with cytosolic N and extracellular C termini) and GPR3 (7 TM helices with extracellular N and cytosolic C termini) from human, VKOR (4 TM helices without cytosolic terminus) and PIP1;2 (6 TM helices, with two cytosolic termini) from *Arabidopsis*. A negative control (Nub^G^) failed to activate reporter genes in combination with all the above proteins, indicating that membrane proteins eliminated auto activation (Fig. 2a to 2d). The positive control (Nub^WT^), however, activated reports genes with three of the proteins, PIP1;2, GPR3 and HPN, due to TA released upon the cleavage by USP (Fig. 2a to 2c). Interestingly, not all TM proteins activated reporter genes even they were combined with Nub^WT^. For example, VKOR, a reported TM protein, can’t activate reporter genes even when it is combined Nub^WT^ (Fig. 2d). Similar results are also found in GPR3 N terminal fusion and HPN C terminal fusion (Fig. 2b, c). The above results demonstrate that membrane proteins without truncation only activate reporter genes with a cytoplasmic termini co-transformed with Nub^WT^.

### Implications of SUT for a transient splicing signal peptide

Having tested the SUT system to distinguish membrane and nucleo-cytoplasmic proteins, we next evaluated the application of the SUT to study released peptides which often occur during the maturation or translocation of nascent proteins. A wheat broad strip rust resistance protein, WKS1, binds membrane lipids and undergoes N terminal cleavage during its transportation into the chloroplast, and thus is a good example to study peptide truncation [4].

**Fig. 3:**
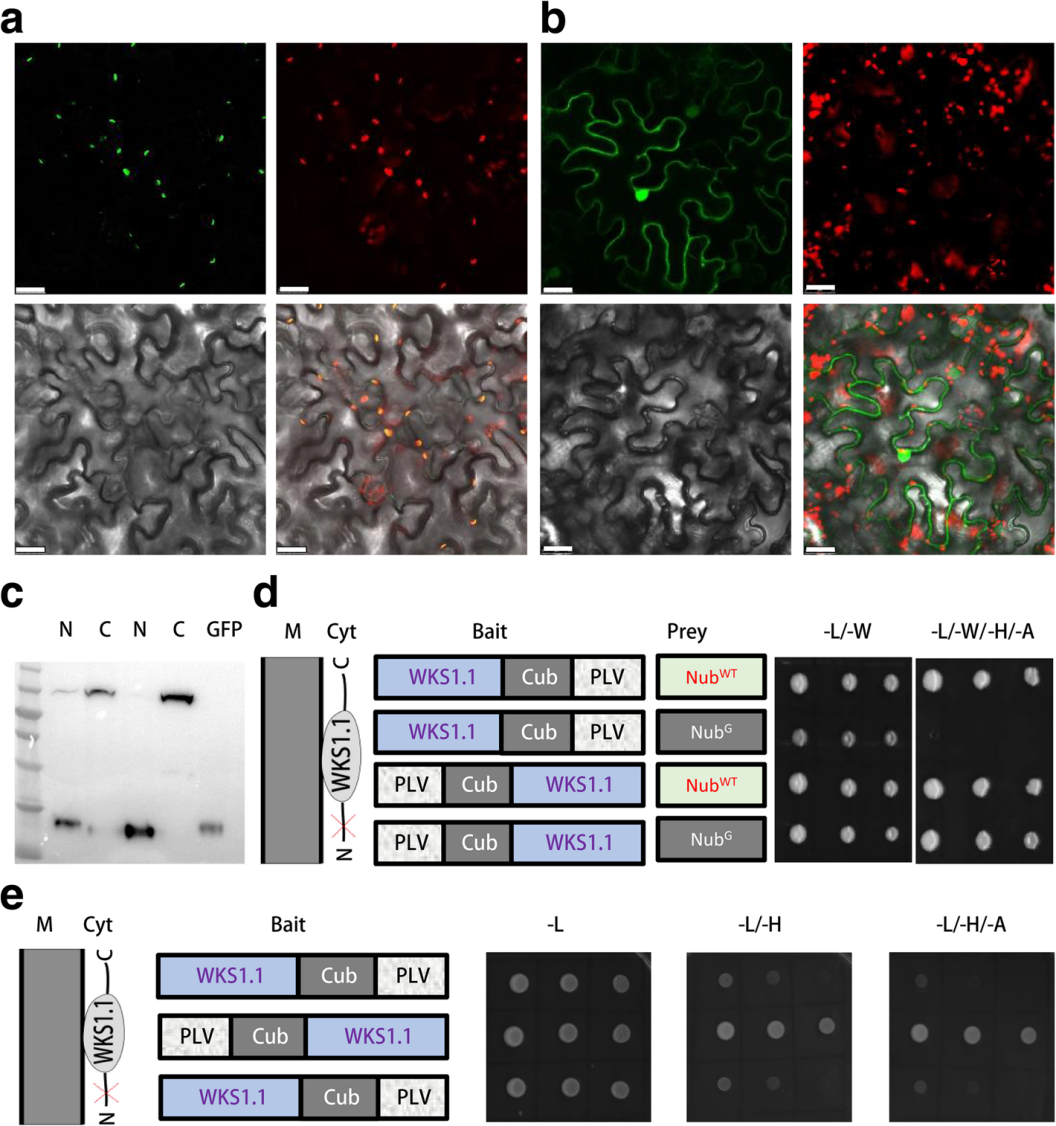
Subcellular localization and performance of SUT on a membrane protein with N terminal released peptide. **a**-**b** Confocal micrographs of WKS1.1-GFP (**a**) or GFP-WKS1.1 (**b**) fusion proteins in tabaco epidermal cells. Bar stands for 25 μm. Green: GFP fusion proteins; red: chloroplast autofluorescence; bright light: cell shape; and overlay of the three panels. **c** Detection of WKS1.1-GFP or GFP-WKS1.1 fusion proteins with anti GFP antibody. N, GFP-WKS1.1; C, WKS1.1-GFP; GFP, empty vector. Duplicate experiments were shown with N and C samples. **d** Growth of yeast cells with WKS1.1 and control plasmids in SUT system. **e** Growth of yeast cells with WKS1.1 in the SUT system

We first validated the truncation in tobacco cells and analyzed the subcellular localization of WKS1-GFP and GFP-WKS1 fusion proteins. Fluorescence signal from WKS1-GFP fusion protein concentrates in chloroplast while signal from GFP-WKS1 disperses in cytosol and also the nucleus (Fig. 3a, b). In accordance, the majority of the WKS1-GFP protein remains intact (around 100 kD) but that of GFP-WKS1 is cleaved (less than 30 kD) (Fig. 3c). These data show that the reporter protein, GFP, is released with the N terminal released peptide of WKS1.

We then analyzed whether SUT system could detect the released peptide with WKS1 as a protein model. In SUT, WKS1 C terminal fusion (WKS1-Cub-TA) activates reporter genes with Nub^WT^ but not Nub^G^, demonstrating that WKS1-Cub-TA fusion protein binds to the plasma membrane (Fig. 3d) as WKS1 binds membrane lipids, PA and PIPs [4]. However, N terminal fusion of WKS1 (TA-Cub-WKS1) activates reporter genes both with Nub^WT^ and Nub^G^, indicating that TA-Cub is released with the cleavage of released peptide in WKS1 (Fig. 3d).

Based on the above results, we further predict that TA-Cub-WKS1 is capable of activating reporter genes without combinations with Nub^WT^ or Nub^G^. Yeast cells with TA-Cub-WKS1 alone grow well on SD/−L/−H/−A medium, indicating that TA-Cub is released with released peptide and the function of ubiquitin is not necessary here (Fig. 3e).

## Discussions

### Comparison with other large scale and computational approaches

The SUT system is designed to study the topology of a single target protein (s) which could originated from a large scale proteome analysis or from prediction in a computational platform based on whole genome data. Large scale proteome analysis of transmembrane topology relies on the efficiencies of labeling and enrichment, which could limit the capture of a particular protein. Despite of the abundant information, the bioinformatics predictions often produce faint results, as shown in Table 1. Thus SUT is developed to work as a preliminary validation of the large scale information. All the vectors in SUT are gateway compatible, which are easy to operate using the commercial LR reaction kit. And SUT is performed in a simple yeast experiment which could be accessed in most labs. A typical SUT experiment can be done within as short as 1 week. Therefore, SUT has advantages of simple and reliable over the large scale approaches.

### Limitations of SUT for released peptides

We demonstrated that SUT could distinguish an N terminal truncated peptide of WKS1. This method alone, however, is supposed to meet trouble in distinguishing membrane proteins with multiple truncation peptides at both N and C termini (2S seed storage protein) from nucleo-cytoplasmic proteins [23]. However, this issue could be addressed if this system is combined with a Western Blot experiment by comparing protein sizes of TAs with the predicted fusion protein molecular mass to see if they are significantly smaller (truncation, e.g. N samples in Fig. 3e) or similar (a real nucleo-cytoplasmic protein).

### Limitations due to heterologous protein production

It’s noteworthy that SUT does not provide solutions for all the proteins. One of the most ambiguous disadvantages of yeast system comes from the protein folding and post-translational modifications. Although the six tested proteins expressed well here (Additional file 1: Figure S1), some target proteins are predicted to fold incorrectly in yeast. In yeast, some proteins may also be toxic or lack necessary post-translational modifications, e.g. phosphorylation, which are important for the subcellular localization or topology of some proteins [24]. In these respects, it is notable that cautious should be taken in the interpretation of preliminary topological information from SUT and host is the one to draw final conclusions.

## Conclusions

The SUT system provides topology information for membrane proteins in a simple yeast experiment. Taking advantages of simple and high accuracy, this system serves as a straightforward experimental approach to fill the gap between computational prediction and crystallization by revealing preliminary topological and extracellular peptides information, which are valuable for pathogen recognitions and new drug developments.

## Methods

### Yeast split ubiquitin system

The destination vector, pMetYC-Dest, and positive/negative control vectors are available at Arabidopsis Research Center (www. arabidopsis.org). The N terminal fusion destination vector, pMetYN-Dest, were constructed in this study by overlapping PCR of each elements from pMetYC-Dest, reorganized and cloned into the same linearized backbone by ligation independent cloning.

The PIP, GPR3, HPN and VKOR coding regions were amplified from RT products (Additional file 2: Table S1), cloned into pDONR207 vector by BP Clonase II for 30 min at RT after and transformed into *E Coli* DH5α cells according to the user manual (Invitrogen, Life Technologies, Carlsbad, CA, USA). WKS1 (WKS1.1) entry clones were reported in an earlier study [4]. GUS gene was supplied within the LR Clonase II reaction kit (Invitrogen). The resulted plasmids were confirmed by sequencing and saved as Entry vectors. The target genes were then cloned into pMetYN-Dest or pMetYC-Dest vectors by LR reactions using LR Clonase II as described in the user manual.

The bait or prey vectors were transformed through the lithium acetate method into yeast strain THY.AP4 or THY.AP5 and selected on SD-L/or SD/−W medium, respectively [11]. Yeast component cell preparation and transformation were described in the Yeast Protocol Handbook (PT3024–1, Clontech Laboratories, Inc., Mountain View, CA, USA). In brief, yeast cells were recovered in an YPDA solid plate (YPDA plus 2% agar) overnight and a single colony were grown overnight in YPDA liquid medium (2% peptone, 1% yeast extract and 0.003% adenine) at 30°C. 10 ml of the overnight cultures were transferred into 100 ml YPDA liquid medium to bring OD_600_ around 0.2 ~ 0.3. The yeast cells were incubated in a 30°C shaker at 230 rpm for 3 h until the OD_600_ reached 0.4 ~ 0.6. The cultures were transferred into sterile 50 ml tubes and centrifuged at 1000 g for 5 min at RT to discard the supernatants. The cell pellets were resuspended in 10 ml sterile water and centrifuged for another 5 min at 1000 g to remove the supernatant. The cells were resuspended in 0.5 ml of fresh sterile 1X TE/LiAc (10 mM Tris-HCl and 100 mM LiAc, pH 7.5). Then 0.1 μg plasmid DNA and 0.1 mg of carrier DNA were added into sterile tubes. 0.1 ml yeast cells were added and mixed well by vortexing. 0.6 ml of sterile PEG LiAc solution (40% PEG in 1 X TE/LiAc) were added into each tube and mixed well by vortexing. The samples were incubated for 30 min in a 30°C shaker set at 200 rpm. 70 μl of DMSO was added into each sample and mixed by gentle inversion. The samples were incubated in a 42°C water bath for 15 min and then chilled on ice for 2 min. The yeast cells were centrifuged at 13,000 rpm for 5 s to remove the supernatant. The cell pellets were resuspended in 100 μl 0.9% NaCl and plated on SD plates.

After validation by PCR, the two haploid yeast strains were mated and selected on SD/−L/−W medium for diploid cells containing both plasmids. Diploids cells were grown in SD/−L/−W or SD/−L/−W/−H/−A plates to test the activation of reporter genes in a 30°C oven for 2 days. WKS1 single transformed yeast cells were grown on SD/−L or SD/−L/−H/−A plates to test the auto-activation of a truncated peptide.

### Plant infiltration and Western Blot

Tobacco, *Nicotiana benthamiana*, was grown in a long day (16 h light/8 h dark) growth chamber set at 22°C. 3-week-old leaves were used for the infiltration experiment. *WKS1* gene was cloned into pMDC43 and pMDC83 plasmids by LR reactions using LR Clonase II (Invitrogen) as described to construct N or C terminal GFP fusion proteins [25]. The resulted plasmids were confirmed by PCR, transformed into *A. tumefaciens* strain GV3101. The cells were incubated at 30°C overnight in a shaker at 200 rpm, harvested by centrifugation at 1500 g for 5 min at RT and resuspended in Agrobacterium infiltration solution (10 mM MgCl_2_, 10 mM of MES and 200 mM acetosyringone) with OD_600_ around 0.8 for 3 h in RT. The cells were infiltrated into abaxial epidermis of tobacco leaves by 1 ml needleless syringes.

Two days later, GFP images were taken using an inverted confocal microscope equipped with a 40X oil-corrected objective in Leica TCS SP8 (Leica, Microsystems, Mannheim, Germany). Images were captured at 488-nm laser excitation and 500-to 550-nm long pass emission filters. 600-nm and over long pass emission filter was used to image the auto fluorescence of chloroplast.

To check the truncation of GFP-WKS1.1 in plant, total proteins were extracted from the same leaves fine powder ground in a high-throughput homogenizer for 15 s at 60 Hz in liquid nitrogen with 1X PBS at 100 mg fresh weight per ml. The protein samples were normalized with equal amounts RuBisCo large unit and then analyzed by Western Blot with anti-GFP monoclonal antibody at 1:1000 dilutions (GFP-Tag (7G9) mAb, Abmart, Shanghai, China). The proteins were detected with goat anti-Mouse IgG-HRP (M21001, Abmart) and then developed by ECL developing system in a Tanon 5200 Imaging System (Tanon Science & Technology Co., Ltd., Shanghai, China).

## Additional files

Additional file 1: Figure S1. Target protein content analysis in yeast cells expressing N or C terminal fusion proteins. N,TA-target; C, target-TA. (JPEG 164 kb)

Additional file 2: Table S1. Primers used in this study. (XLSX 9 kb)

## Abbreviations

SUT: Split ubiquitin topology analysis
TA: Transcription activator (LexA-VP)
TM: Transmembrane
TP: Truncated peptide
WKS1: Wheat Kinase START1
