# Supplementary figures and images for: A split ubiquitin system to reveal topology and released peptides of membrane proteins
Source: BMC Biotechnol. 2017 Sep 2;17:69. doi: 10.1186/s12896-017-0391-0 (PMC5581432; doi:10.1186/s12896-017-0391-0)

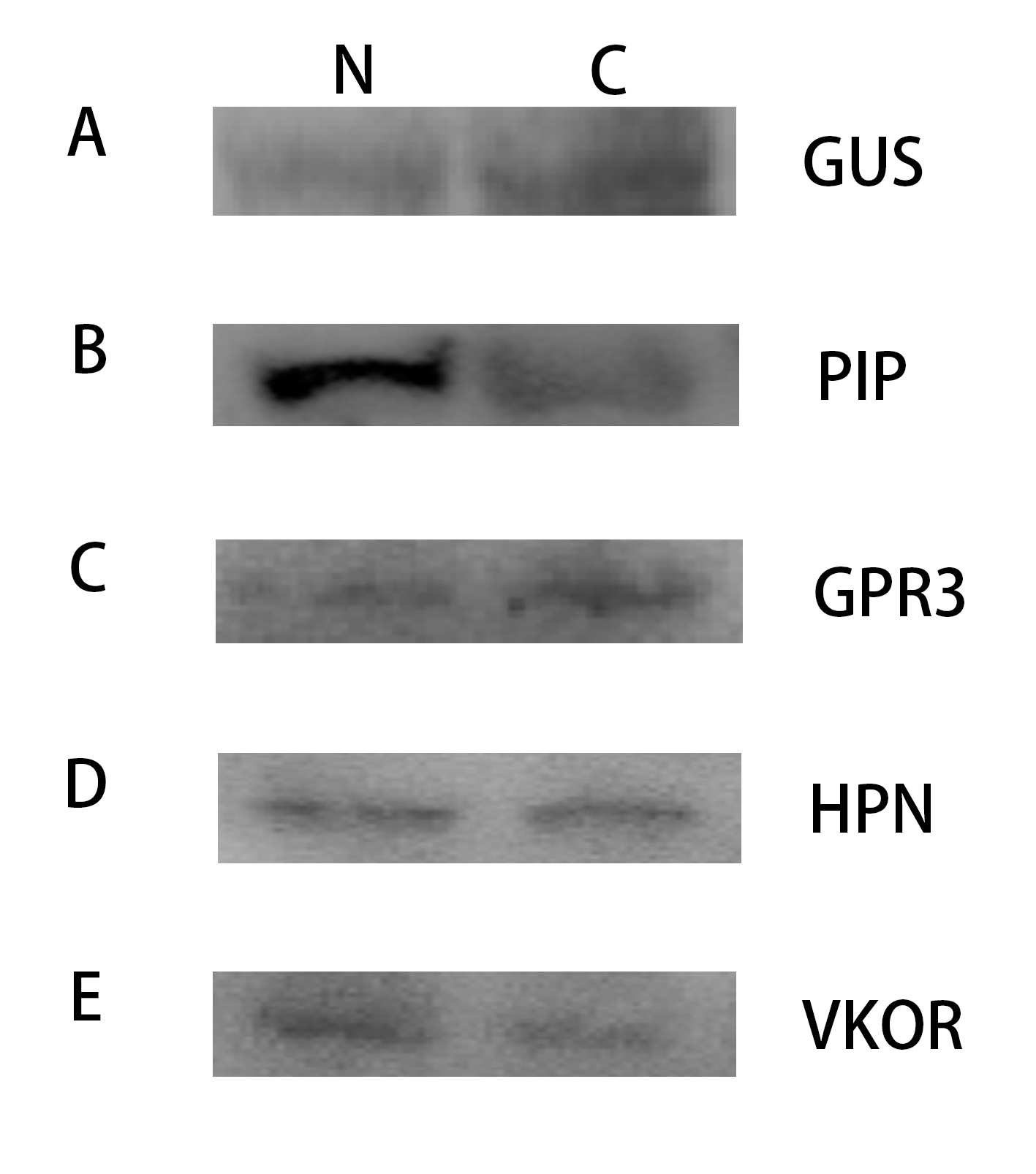

Supplement: Supplementary file 1 — Target protein content analysis in yeast cells expressing N or C terminal fusion proteins. N,TA-target; C, target-TA. (JPEG 164 kb) [file 12896_2017_391_MOESM1_ESM.jpg]
